# Formulation and Characterization of Solid Lipid Nanoparticles Loading RF22-c, a Potent and Selective 5-LO Inhibitor, in a Monocrotaline-Induced Model of Pulmonary Hypertension

**DOI:** 10.3389/fphar.2020.00083

**Published:** 2020-02-28

**Authors:** Angela Liparulo, Renata Esposito, Debora Santonocito, Alejandra Muñoz-Ramírez, Giuseppe Spaziano, Ferdinando Bruno, Jianbo Xiao, Carmelo Puglia, Rosanna Filosa, Liberato Berrino, Bruno D'Agostino

**Affiliations:** ^1^ Department of Experimental Medicine, Section of Pharmacology “L. Donatelli,” University of Campania “L. Vanvitelli,” Naples, Italy; ^2^ Department of Drug Sciences, University of Catania, Catania, Italy; ^3^ Departamento de Ciencias del Ambiente, Facultad de Química y Biología, Universidad de Santiago, Casilla, Correo, Chile; ^4^ Institute of Food Safety and Nutrition, Jinan University, Guangzhou, China; ^5^ Department of Environmental Biological and Pharmaceutical Sciences and Technologies, University of Campania “L. Vanvitelli,” Naples, Italy; ^6^ Consorzio Sannio Tech-AMP Biotec, Apollosa, Italy

**Keywords:** pulmonary arterial hypertension, solid lipid nanoparticles, 5-lipoxygenase, inflammation, monocrotaline, RF-22c, leukotrienes

## Abstract

Pulmonary arterial hypertension (PAH) is a rare but fatal disease characterized by persistent elevated blood pressure in the pulmonary circulation, due to increased resistance to blood flow, through the lungs. Advances in the understanding of the pathobiology of PAH clarify the role of leukotrienes (LTs) that appear to be an exciting new target for disease intervention. Over the years, our group has long investigated this field, detecting the 1,2-benzoquinone **RF-22c** as the most powerful and selective competitive inhibitor of the enzyme 5-lipoxygenase (5-LO). With the aim to improve the bioavailability of RF-22c and to confirm the role of 5-LO as therapeutic strategy for PAH treatment, we developed a solid lipid nanoparticle (SLN) loaded with drug. Therefore, in monocrotaline (MCT) rat model of PAH, the role of 5-LO has been investigated through the formulation of RF-22c-SLN. The rats were randomly grouped into control group, MCT group, and MCT + RF22-c group. After 21 days, all the animals were sacrificed to perform functional and histological evaluations. RF22-c-SLN treatment was able to significantly reduce the mean pulmonary arterial pressure (mPAP) and precapillary resistance (R-pre) compared to the MCT group. The MCT induced rise in medial wall thickness of pulmonary arterioles, and the cardiomyocytes width were significantly attenuated by RF22-c-SLN formulation upon treatment. The results showed that the selective inhibition of 5-LO improved hemodynamic parameters as well as vascular and cardiac remodeling by preventing induced pulmonary hypertension. The improved sustained release properties and targeting abilities achieved with the innovative nanotechnological approach may be therapeutically beneficial for PAH patients as a consequence of the increase of pharmacological effects and of the possible reduction and/or optimization of the drug frequency of administration.

## Introduction

The World Health Organization defines pulmonary arterial hypertension (PAH) as a rare but fatal disease (Rabinovitch, 2012; Corris and Degano, 2014; Fallah, 2015) characterized by persistent elevated blood pressure in the pulmonary circulation, due to increased resistance to blood flow, through the lungs (Montani et al., 2013).

The increased pulmonary vascular resistance in PAH has been attributed to two major factors, vasoconstriction and remodeling of the vascular wall. It has been observed that in some patients, inflammation appears to play a major pathogenic role (Nicolls et al., 2005; Hassoun et al., 2009), activation of inflammatory cells and increased production of their mediators are important features of PAH (Price et al., 2012). In fact, higher circulating levels of monocyte chemoattractant protein 1 (MCP-1), tumor necrosis factor α (TNF-α), and interleukins in patients with idiopathic PAH than in healthy controls are involved (Kimura et al., 2001; Itoh et al., 2006; Sanchez et al., 2007; Zabini et al., 2014; Sharma et al., 2016). Macrophages, in particular, are prominent components of the inflammatory infiltrates in the lungs of patients and animals with PAH (Tuder and Voelkel, 1998; Dorfm̈ller et al., 2003; Taraseviciene-Stewart et al., 2007; Tamosiuniene et al., 2011). Recent evidence demonstrates that leukotrienes (LTs), important eicosanoid products of leukocytes, including macrophages, play an essential role in the inflammatory mechanism in PAH (Bittleman and Casale, 1995): in patients with PAH, it was highlighted that the level of 5-lipoxygenase (5-LO) expression is increased in pulmonary macrophages and small pulmonary artery endothelial cells (PAECs) (Wright et al., 1998). Moreover, it was observed that targeted disruption of the 5-lipo-oxigenase (5-LO) gene in mice and pharmacologic blockade of 5-LO function in rats reduce chronic hypoxia-associated PAH (Voelkel et al., 1996; Song et al., 2005; Tian et al., 2013). To these notions, it is possible to add that LTs, able to promote inflammatory processes in many diseases, are implicated in the progression of PAH (Mori and Beilin, 2004).

Taken together, these data give evidence of major involvement of inflammation mediators and their synthesizing proteins (such as LTs synthetized by 5-LO) in PAH and make them attractive pharmacological targets to counteract PAH pathology (Sharma et al., 2016). Within the context of our investigations toward the synthesis of different compound libraries with prospects for therapeutic use, we recently studied the natural and synthetic quinone derivatives. Over the years, our group has long investigated about the 1,4-benzoquinones class as lead compounds having potent antioxidant, anti-inflammatory, and anticancer properties and highlighted their efficient inhibitory activity against 5-LO (Filosa et al., 2013; Petronzi et al., 2013; Schaible et al., 2014; Filosa et al., 2015; Zappavigna et al., 2016).

**Figure 1:**
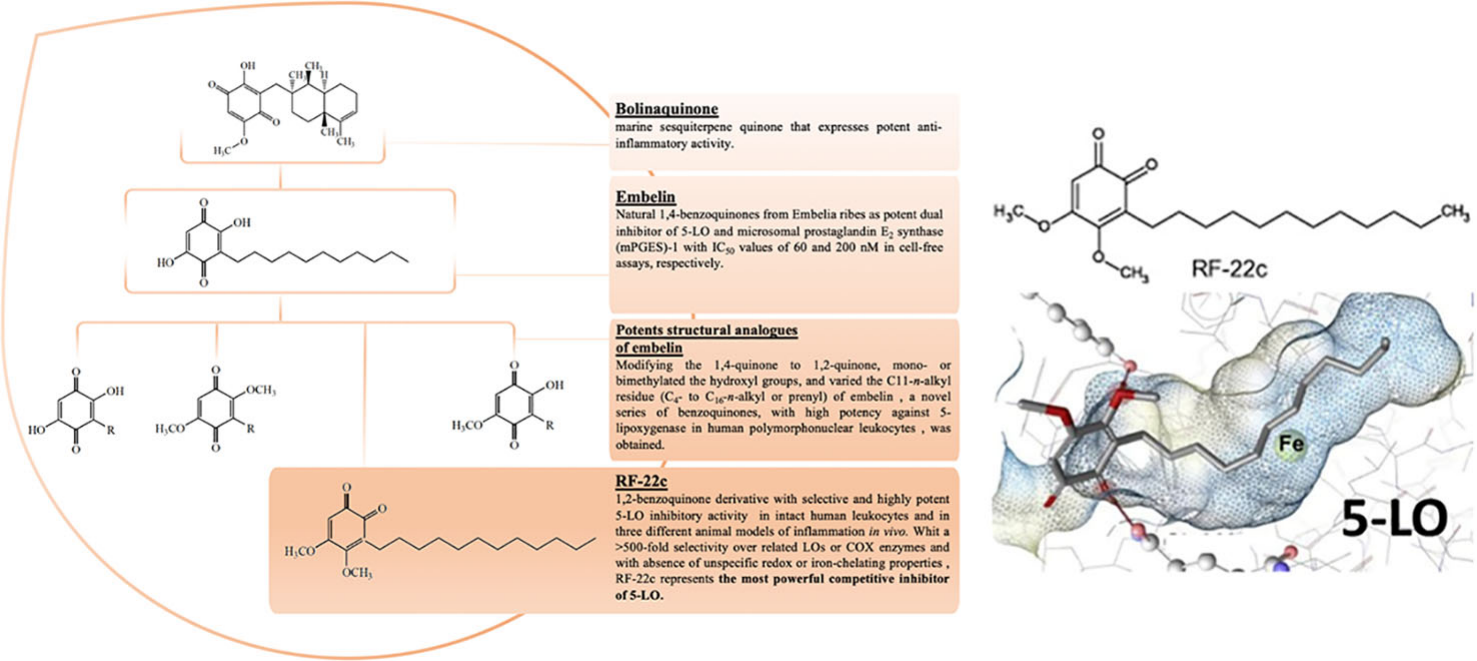
Drug development process of RF22-c.

In detail, in our previous study aiming to assess the biological activity of natural and synthetic benzoquinone derivatives (Petronzi et al., 2011), we detected the natural product *embelin* as potent dual inhibitor of 5-LO and microsomal prostaglandin E2 synthase (mPGES)-1 with IC50 values of 60 and 200 nM in cell-free assays, respectively (Schaible et al., 2013). Moreover, further modification of the structure, to improve the inhibitory potential as well as to investigate structure–activity relationships (SARs), led to the discovery of 3-dodecyl-4,5-dimethoxy-1,2-benzoquinone (**RF22-c**). It was demonstrated that **RF22-c** potently inhibited LT formation in cellular and blood assays, and on the molecular level, the 1,2-benzoquinone inhibited the 5-LO with a competitive mechanism, without any unspecific redox or iron-chelating properties, affirming itself as the most highly selective and potent 5-LO inhibitor actually discovered, exhibiting >500-fold selectivity over related lipoxygenases (LOXs) or cyclooxygenase (COX) enzymes (Schaible et al., 2016; Peduto et al., 2017; Bruno et al., 2018) (**Figure 1**). The advent of nanotechnology has shown highly significant potential in the delivery of a wide range of therapeutic agents (Puglia et al., 2010; Goethals et al., 2013; Puglia et al., 2018). Solid lipid nanoparticles (SLNs) in particular have emerged as an effective and promising alternative to conventional drug delivery systems. They are colloidal particles of submicron size, with a diameter between 50 and 1,000 nm. They are formed from a single solid lipid at physiological temperature, stabilized by the surfactant. Besides, SLNs have revolutionized the release of drugs, allowing therapeutic agents to be selectively directed to a specific organ. The encapsulation of the drugs into these nano-carriers increases their solubility, bioavailability, and cell uptake. A key role is played by the nature of the drug to be incorporated since it has to be solubilized in the lipid matrix in order to have a good entrapment efficiency.

The aim of this work was to develop a nanotechnology-based formulation containing **RF22-c** as active pharmaceutical ingredient (API). RF22-c**-**loaded SLNs were characterized *in vitro* for their mean size, zeta potential, population homogeneity, and morphology, while the effectiveness of **RF22-c** on PAH progression, when embedded in SLN, was evaluated in an *in vivo* model.

## Materials and Methods

### Drug Preparation and Treatment

RF22-c was synthesized and chemically analyzed with purity of 99% determined by spectroscopic and analytic methods as reported previously (Filosa et al., 2013). All reagents were analytical grade and purchased from Sigma-Aldrich (Milano, Italy). Carlo Erba silica gel 60 was used for Flash chromatography (230–400 mesh; Carlo Erba, Milan, Italy). Thin-layer chromatography (TLC) was performed using plates coated with silica gel 60 F254 nm purchased from Merck (Darmstadt, Germany). 1H and 13C NMR spectra were registered on a Bruker AC 300. Chemical shifts are shown in parts per million (ppm). Combustion analysis was chosen to assess the purity (95% or higher) of all final products that were evaluated for bioactivity (using a Carlo Erba 1106 elemental analyzer) (Filosa et al., 2015). The test compounds were dissolved in dimethyl sulfoxide (DMSO) and stored in the dark at -20°C; freezing/thawing cycles were kept to a minimum.

### SLN Formulation

RF22-c-loaded SLNs were prepared by solvent-diffusion technique (Brugè et al., 2013). Softisan 100 (0.023 g) and RF22-c (7 mg) were solubilized in ethanol (4.6 ml) and melted at 45°C. The aqueous phase was constituted by hydroxypropyl methylcellulose (0.23 g), soy lecithin (0.23 g), Lutrol F68 (0.23 g), and distilled water (23 ml). The melted lipid phase was added dropwise to the hot aqueous phase (50°C) by using a high-speed stirrer (Ultra-Turrax T25, IKA-Werke GmbH & Co. Kg, Staufen, Germany) at 15,000 rpm for 8 min, maintaining the temperature of at least 10°C above the lipid melting point.

The obtained pre-emulsion was ultrasonified by using a Labsonic 2000 (B. Braun, Melsunen, Germany) for 5 min (0.5 cycle). Then, the hot dispersion was cooled in an ice bath for 5 min. Finally, the organic solvent was removed by vacuum.

### SLN Characterization

The average size and zeta potential were determined by photon correlation spectroscopy (PCS) using a Zeta Sizer Nano-ZS90 (Malvern Instrument Ltd., Worcs, England). The experiments were carried out using a 4.5-mW laser diode operating at 670 nm as light source; size measurement was carried out at a scattering angle of 90°. The samples were diluted (1:10) in distilled water. The measurements were determined at 25°C in triplicate. During the experiment, refractive index of the samples always matched the liquid (toluene) to avoid stray light (Puglia et al., 2016).

The zeta (*ξ*) potential was determined from electrophoretic mobility through the Smoluchowski equation (Equation 1):

(1) $$\vartheta =\left(\frac{\epsilon \cdot E}{\eta }\right)\xi$$

ϑ is the measured electrophoretic velocity, *η* is the viscosity, *ϵ* is the electrical permittivity of the electrolytic solution, and *E* is the electric field. The accuracy was 0.12 μm cm/Vs for aqueous systems.

### Scanning Electron Microscopy

Scanning electron microscopy (SEM) analysis was performed on an FEI Quanta 200 SEM (Eindhoven, Netherlands) at 5–20 kV acceleration voltage with a secondary electron detector. Before the analysis, a drop of each dispersion was deposited onto aluminum SEM stubs and let to evaporate at room temperature. Therefore, the samples were sputter-coated with a 10-nm thick gold-palladium alloy.

### Determination of Drug Loading

The percentage of RF22-c encapsulated in the lipid system was calculated in the following way: a stationary amount of SLN dispersion was filtered *via* a Pellicon XL tangential ultrafiltration system (Millipore, Milan, Italy) supplied by a polyethersulfone Biomax 1000 membrane (Millipore, Milan, Italy) with a 1,000,000 Da molecular weight cutoff. An amount of withheld material was lyophilized, solubilized in dichloromethane, and parsed from UV spectrophotometry at 242 nm (Lambda 52, PerkinElmer, MA). Calibration curves for the validated UV assays of RF22-c were run on six solutions in the concentration range 10–100 µg/ml. The correlation coefficient was >0.99. Each point represented the average of three measurements, and the error was calculated as standard deviation (± SD).

RF22-c incorporation efficiency was explicit as drug recovery (DR) and calculated using the following Equation (2):

(2) $$Drug\;recovery\;\left(\%\right)=\frac{Mass\;of\;active\;in\;SLN}{Mass\;of\;active\;fed\;to\;the\;system}\times 100$$

Possible lipid interferences during UV determination of RF22-c were also investigated by matching the two standard curves of the substance alone and in the presence of lipids.

The differences observed between the standard curves were within the experimental error, thus inferring that no lipid interference occurred (data not shown).

### *In Vitro* Release Study

The amount of RF22-c released from SLN was calculated as reported elsewhere (Puglia et al., 2012). Briefly, SLNs containing RF22-c equivalent to 30 µg/ml were dispersed in phosphate buffer solution (pH 7.4) and then divided into different aliquots in glass vials. The vials were placed in a water bath which maintained a constant temperature of 37°C. Since solubility of RF22-c in aqueous solutions is extremely low, this remained in the form of crystals in the releasing medium. At set intervals, the batch was centrifuged at 3,000 rpm for 10 min to divide the released drug in crystal form from the dispersion of SLN. After centrifugation, the sample was recovered and analyzed by UV spectrophotometer at 242 nm to determine the RF22-c amount.

### Animal Study

The experimental protocol was approved by the Animal Care and Use Committee of the University of Campania “Luigi Vanvitelli” (294/2016-PR 24.03.2016). Animal care complied with Italian regulations on the protection of animals used for experimental and other scientific purposes (116/1992) as well as with the EU guidelines for the use of experimental animals (2010/63/EU) (Cappetta et al., 2018).

The experiments were performed on 30 outbred Wistar male rats (Charles River Laboratories, Lecco, Italy), body mass 200–300 g, 5 weeks old, housed in the Animal Facility of the University of Campania “Luigi Vanvitelli,” in standard cages, two animals per cage.

Food and water were supplied *ad libitum*. Room temperature was set at 22°C–24°C, relative humidity at 40%–50%, and the day/night cycle at 12 h/12 h. Rats were acclimated for 7 days before initial treatment. All efforts were made to minimize animal suffering and reduce the number of animals used in the experiments.

Monocrotaline (MCT, Sigma, Germany) was made at a concentration of 60 mg/ml by dissolving it in HCl-acidified Dulbecco's phosphate-buffered saline (PBS) and adjusted the pH to 7.2 with NaOH.

### Experimental Design

The rats were randomly grouped as follows: *control group*, receiving vehicle at day 0 and sacrificed after 21 days (n = 10); *MCT group*, rats receiving a single subcutaneous injection of MCT (60 mg/kg) at day 0 and sacrificed after 21 days (n = 10); *MCT + RF22-c group*, rats receiving a single subcutaneous injection of MCT (60 mg/kg) at day 0 and immediately treated with an intraperitoneal administration of RF22-c-loaded SLNs (10 mg/kg) daily for 5 days and sacrificed after 21 days (n = 10). The rats were sacrificed to perform functional evaluations, and sampling of pulmonary and cardiac tissue was performed for morphometric measurements. Throughout the 21 days of the experimental protocol, no difference in body weight between the groups was recorded, and it was not registered any animal death (Tabata et al., 1997; Tong et al., 2018).

### Hemodynamic Studies

The animals were anesthetized with ketamine (100 mg/kg) and medetomidine (0.25 mg/kg) administered intraperitoneally. The surgery was performed at the 21st day. The rats were placed in dorsal position on the operation table. The registration of hemodynamic parameters in rats was performed by using a Hugo Sachs Electronik Haemodyn (Harvard Apparatus GmbH, Germany) (Uhlig and Wollin, 1994; Uhlig et al., 1995). Through this exclusive system, it is possible, with direct measurements, to obtain *arterial blood pressure (PA)* and *pulmonary venous pressure (PV)* and *flow (Q).* For the determination of precapillary (Rpre) and postcapillary (Ppost) resistance, the measurement of the microvascular pressure (Pc) is required. Pc is determined by the double occlusion technique (Hakim et al., 1996; Singh et al., 2016).

Double occlusion (DO) was performed by means of two solenoid pinch valves (response time 10–30 ms, Anderson, Dortmund, FRG) mounted before and after the lung that was simultaneously clamped by an electronic trigger.

Segmental vascular resistances were calculated according to

$$R_{pre}=\;\frac{P_{A}-\;P_{c}}{Q}\text{ and }R_{post\;}=\frac{Pc-PV}{Q}$$

### Remodeling of Pulmonary Arterioles

The lung tissue immersed in 10% formalin was embedded in paraffin and cut into slices with a thickness of 5 µm; subsequently, it was stained with hematoxylin–eosin.

The images of the transversely cut pulmonary arteries were digitally captured at 40× using an Olympus IX70 microscope and analyzed by the CellSens Standard software. The pulmonary arterioles with a diameter less than 150 µm were selected for morphological analysis performed by a system ImageJ analysis software (National Institutes of Health, Inc., Bethesda, MD) (Cui et al., 2009). The wall thickness index (WI) and the arteriolar area ratio index (AI) were assessed as follows:

$$\text{WI}:\;\frac{\left(\text{vascular outer diameter }–\text{ lumen diameter}\right)}{\left(\text{vascular outer diameter }\times \;100\right)\;}$$

$$\text{AI}:\;\frac{\left(\text{vascular cross}-\text{sectional area }–\text{ lumen cross}-\text{sectional area}\right)}{\text{vascular cross}-\text{sectional area }\times \;100}$$

### Cardiomyocyte Morphometry

Hearts from the treated rats were harvested at day 21 to evaluate the degree of right ventricle (RV) hypertrophy, the cross-sectional area of cardiomyocytes has been evaluated in order to assess the enlargement of those ones. Light microscopic examination (×200) was chosen to analyze 50 intact rod-shaped cardiac myocytes. Images of the stained isolated myocytes were digitized, and measurements consisting of unloaded, unstressed cell length, cell width, sarcomeric length, and sarcomere number were obtained through ImageJ analysis software (National Institutes of Health, Inc., Bethesda, MD). Corrupted cardiomyocytes detected based on broken or fragmented cell membranes were not analyzed. This exclusion criterion ensured highly reproducible results with insignificant variation between observers. Moreover, to confirm the cellular dimensions, cardiomyocyte cross-section dimensions were measured in 5-μm RV cross sections stained with hematoxylin–eosin in multiple regions judged to be cut perpendicular to the long axis of the cells by the nearly round shape of capillaries in the region. Cardiomyocyte width was defined by digitizing myocyte cross-sectional areas, and long and short axis cell widths were averaged to obtain mean cardiomyocyte diameter in 50 cells per animal (Du et al., 2010).

### Statistical Analysis

Data are presented as mean ± SEM from animals grouped together by experimental condition. All normally distributed data were analyzed by one-way analysis of variance (ANOVA) followed by Bonferroni *post hoc* test adjustment for multiple comparisons. Posttest adjustments take into account potential error introduced as a consequence of multiple comparisons. GraphPad Prism was used for all statistical analysis. Values of p < 0.05 were considered significant.

## Results

### SLN Formulation and Characterization

RF22-c-loaded SLN was formulated using a valid and highly reproducible method using Softisan 100 (Hydrogenated Coco-Glycerides) as lipid matrix and Lutrol F68 (Poloxamer 188) as surfactant. The lipid matrix most suitable for the formulation was the hydrogenated coconut-glycerides because they are characterized by a low melting point (35°C); this guarantees to the formulation to avoid thermal stress conditions. Furthermore, the formulation strategy we utilized gave interesting results as confirmed by PCS analysis and drug loading determination. SLN showed a mean diameter of 233 ± 0.03 nm, a polydispersity index (PDI) value around 0.28 ± 0.02 and a DR% of 84.3. For zeta potential values, we obtained -23.8 mV for the RF22-c-loaded SLNs, predicting a good long-term stability for the formulation.

**Figure 2:**
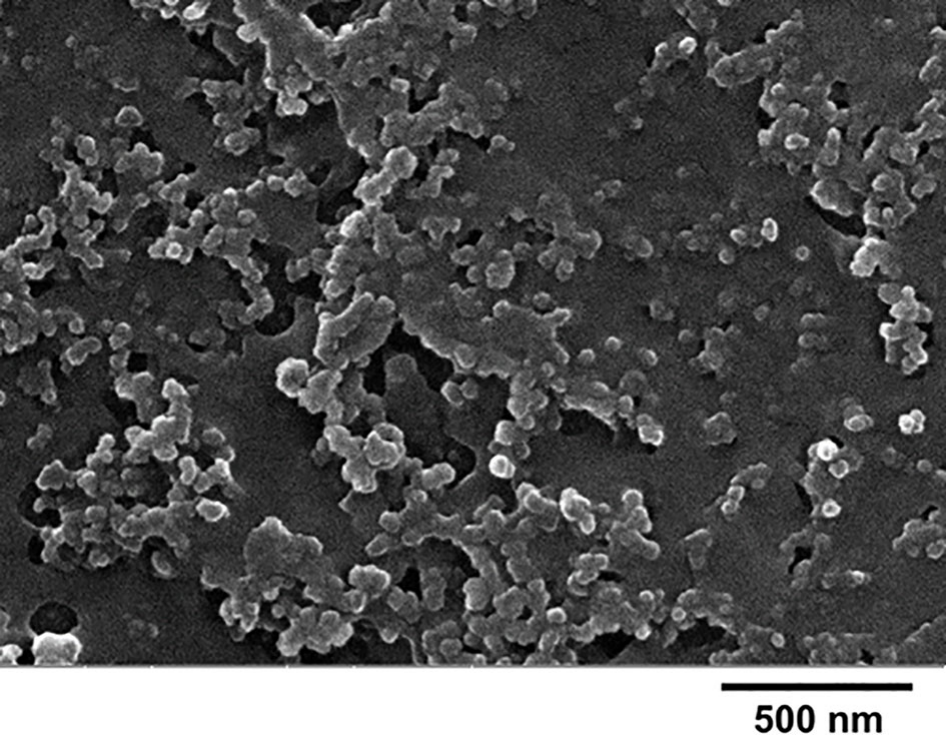
Scanning electron microscopy of RF22-c–loaded solid lipid nanoparticles (SLNs).

Electron microscopy showed a particle size compatible with the one estimated by photon correlation spectroscopy data, and particle shape appears close to the spherical one (**Figure 2**).

**Figure 3:**
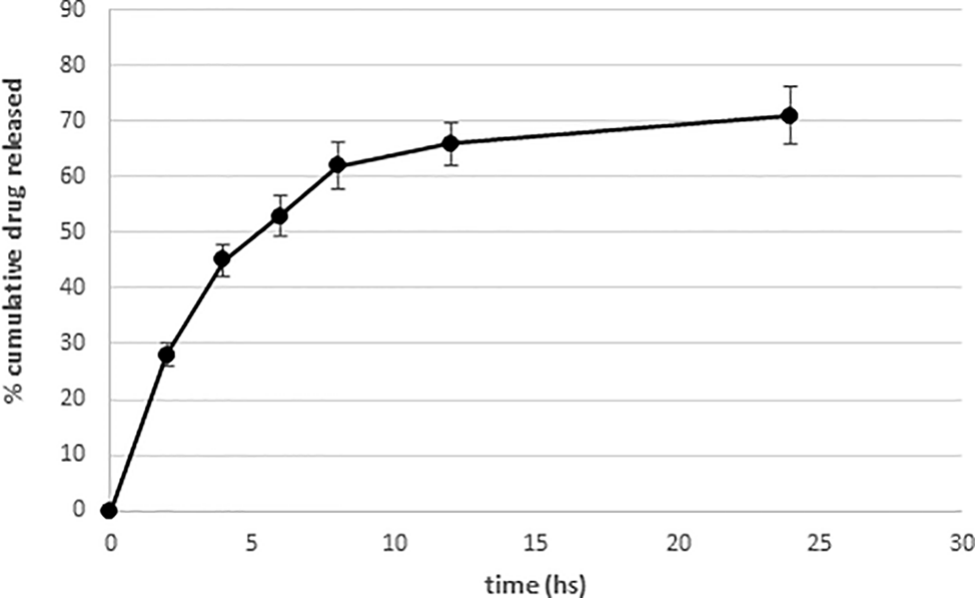
*In vitro* release profiles of RF22-c from solid lipid nanoparticle (SLN). Each point represents the mean value ± SD (n = 3).

The *in vitro* release profile of RF22-c from SLN, as shown in **Figure 3**, follows a “two-step drug release” related to distribution of active compound in SLN matrix (Geszke-Moritz and Moritz, 2016), while the total amount of drug released from the vehicle was about 71% of the loaded drug. With regard to the mechanism, we hypothesized that in the first phase, the drug spread on the surface and the drug that is released immediately because of a wide diffusion in concentration gradient represents the burst release. The second phase and the following prolonged release could be due to the slow release of the drug from the lipid core.

### Hemodynamic Studies

**Figure 4:**
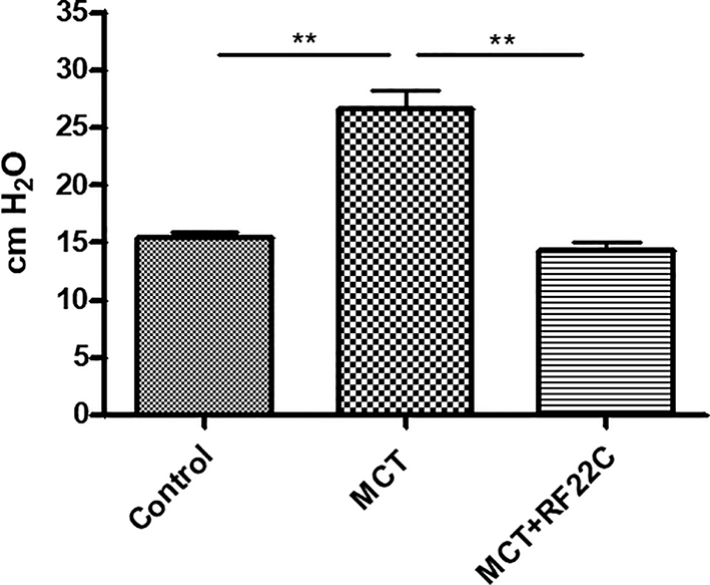
Effect of 5-lipoxygenase (5-LO) inhibition on mean pulmonary arterial pressure (mPAP). Monocrotaline (MCT) significantly enhanced mPAP compared to the control group. In the MCT + RF22-c group, the mPAP values were significantly reduced with respect to MCT group. Data are means ± SE (n = 5); one-way ANOVA followed by Student's t-test was used. (**P < 0.01 MCT vs. control group and MCT + RF22-c).

Hemodynamic measurements show a statistically significant increase (P < 0.001) in mean pulmonary arterial pressure (mPAP) in the MCT group (26,6 cm H_2_O) compared to the control group (15,44 cm H_2_O); after treatment with RF22-c, the mPAP values were significantly reduced to a value comparable to those of the control group (mPAP-RF22-c 13,44 cm H2O, P < 0,001) (**Figure 4**).

**Figure 5:**
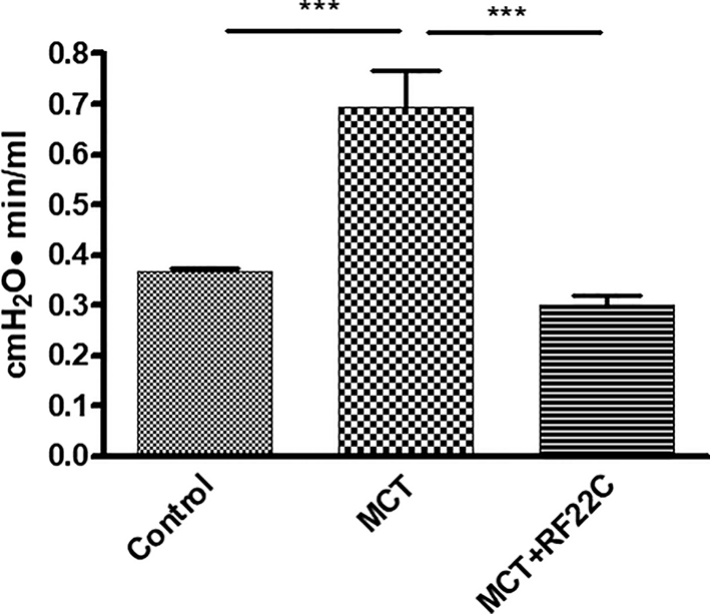
Effect of 5-lipoxygenase (5-LO) inhibition on precapillary resistance (R-pre). Monocrotaline (MCT) significantly enhanced R-pre compared to the control group. In the MCT + RF22-c group, the R-pre values were significantly reduced with respect to MCT group. Data are means ± SE (n = 5); one-way ANOVA followed by Student's t-test was used. (***P < 0.001 MCT vs. control group and MCT + RF22-c).

In addition, vascular resistances show a significant increase in the Rpre in the monocrotaline group compared to the control group, that was significantly decreased after treatment with RF22-c. These hemodynamic values may reflect the effect of RF22-c on the pulmonary arterioles remodeling (**Figure 5**).

### Remodeling of Pulmonary Arterioles and Cardiomyocyte Morphometry

**Figure 6:**
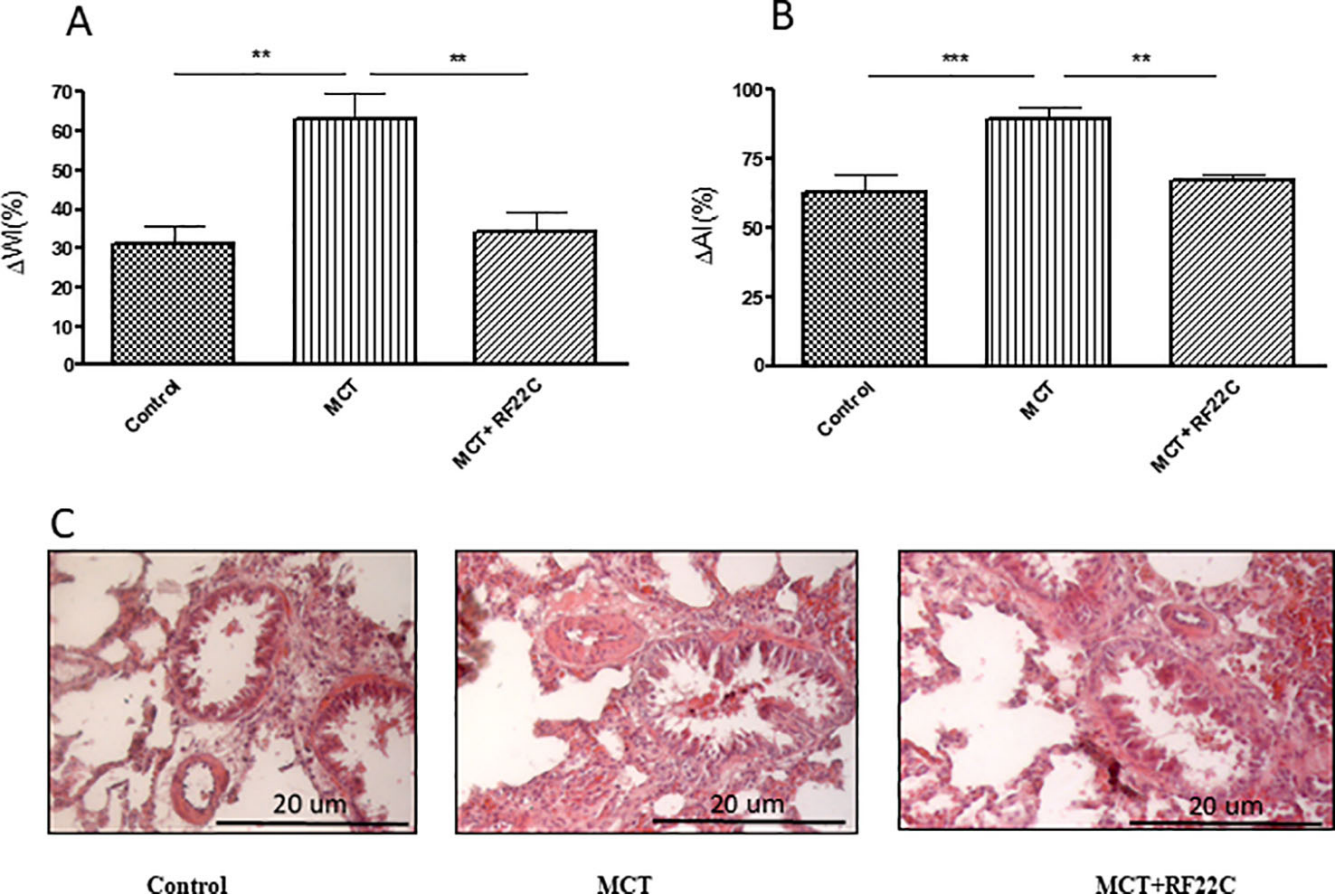
Effects of 5-lipoxygenase (5-LO) inhibition on remodeling of the pulmonary arterioles. Monocrotaline (MCT) significantly enhanced wall thickness index [ΔWI (%)] and arteriolar area ratio index [ΔAI (%)] compared to the control group. In the MCT + RF22-c group, the ΔWI and ΔAI values were significantly reduced with respect to MCT group **(A, B)**. Pulmonary arterioles of control rats, MCT-treated rats, and RF22-c-SLN-treated rats **(C)**. Data are means ± SE (n = 5); one-way ANOVA followed by Student's t-test was used. (***P < 0.001; **P < 0.01).

In the monocrotaline group, WI (62,92%) and AI (89,43%) were significantly increased compared with control group (30,86% and 62,84%, respectively, P < 0,01). RF22-c significantly reduces the increase in WI and AI induced by MCT (34,32% and 67,22% respectively;

P < 0,01) (**Figures 6A–C**).

**Figure 7:**
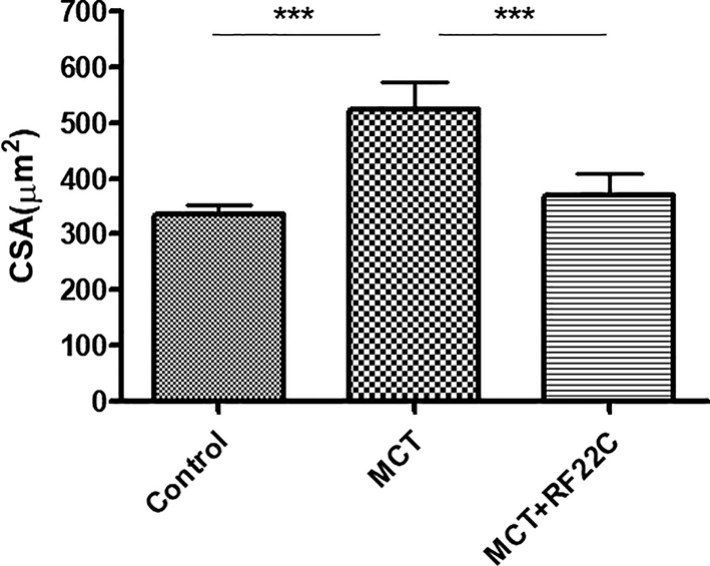
Effect of 5-lipoxygenase (5-LO) inhibition on right ventricle (RV) cardiomyocytes cross-sectional area (CSA). Monocrotaline (MCT) significantly enhanced CSA compared to the control group. In the MCT + RF22c group, the CSA values were significantly reduced with respect to MCT group. Data are means ± SE (n = 5); one-way ANOVA followed by Student's t-test was used. (***P < 0.001 MCT vs. control group and MCT + RF22c).

Moreover, the animals treated with MCT (523.28 µm^2^) show a significant increase of cross-sectional area than the control group (334,44 µm2; P < 0,001); upon treatment with RF22-c the cardiomyocytes width has been significantly reduced (368,55 µm^2^; P < 0,001) (**Figure 7**).

## Discussion

Inflammation in PAH and the clear involvement of different types of inflammatory mediators in the disease progression are well known in literature (Stenmark et al., 1985; Marsh et al., 2018). In this context, we have examined the role of 5-LO in PAH development through the use of RF22-c, a direct inhibitor of the enzyme, in MCT rat model of PAH.

Low solubility of the active quinone is considered the primary obstacle that hinders its usage in pharmaceutical formulations. This is due because the medium at which drugs work is mainly aqueous and low solubility in aqueous media will drastically reduce the drug concentration inducing a poor bioavailability. Nanoparticles represent an alternative medium for hydrophobic drugs to be solubilized in, and carried out through the body to the targeted tissue. The extensively small sizes of nanoparticles give large surface to volume ratio and so many water molecules can surround the particles, enhancing the solubility of hydrophobic compound (Brugè et al., 2013). In this context, SLN played a crucial role in increasing the pharmacological activity of the quinone, in fact RF22-c-loaded SLN, showed a significant reduction of pulmonary pression increased by MCT, bringing the mPAP and precapillary resistance (R-pre) values back to levels comparable to control group. This result could be explained on one hand by the intrinsic selectivity of RF22-c in inhibiting the enzyme 5-LO and on the other by the release profile of the drug from SLN which appears to be sustained, as observed by *in vitro* evidences. We hypothesized that SLNs, by releasing RF22-c in a controlled fashion, were able to increase the amount of drug on the PAH sites with a consequent significant improvement of pulmonary vascular remodeling and pulmonary hypertension and a further increase of therapeutic effects. Recently, a similar mechanism has been documented by Ishihara et al. (2015) in a work regarding the effect of beraprost sodium encapsulation in polymeric nanoparticles on drug pharmacological activity in animal model of PAH.

The reduction of hemodynamic parameters could reflect the significant improvement in vascular remodeling induced by RF22-c treatment. Indeed, increased artery pulmonary smooth muscle cell contractility and then narrowing of pulmonary arteriole lumen could explain the increase of PAP and rise of R-pre in MCT-induced PAH (MCT-PAH). Several studies on animal model of PAH (Meyrick et al., 1980; Nogueira-Ferreira et al., 2015) documented medial thickening as a consequence of proliferation, migration, and hypertrophy of medial smooth muscle cells in small pulmonary arteries (Jones et al., 2004). Here, we observed a remarkable thickening of pulmonary vessel walls and a narrowing of the lumen following MCT treatment. The extent of such morphological variations measured by WI and AI was much lower in the RF22-c group. Therefore, RF22-c partly improved the damaged vascular contractile function and the endothelial dysfunction induced by MCT in pulmonary artery. These structural changes affect not only architecture of the walls of pulmonary arteries but also the heart structure. The significant elevation in PAP, the increasing of RV afterload, and decreasing of endurance induced right ventricular hypertrophy and subsequently right ventricular failure (RVF). A growing number of evidences support the leukotrienes implication in cardiomyocytes' remodeling (Qian et al., 2015), demonstrating a significant reduction in the degree of hypertrophy of the RV and right ventricular systolic pressure (RVSP) induced by agents that affect lipoxygenase metabolite production (Al-Husseini et al., 2015). In our study, the pressure decrease induced by RF22-c treatment resulted in a reduction of heart overload and RV hypertrophy. In fact, through the histologic analysis of RV tissue from rats with MCT-PAH, we documented a decrease in the cardiomyocytes width, suggesting a critical role of 5-LO in the progression of disease in this model.

## Conclusions

Currently, the drug treatments available for the treatment of PAH are ineffective due to the poor understanding of its pathophysiology. Our data have given evidence of a significant involvement of 5-LO in PAH, showing that the selective 5-LO inhibition with RF22-c administered by nanoparticle system significantly reduced pathological parameters, partly preventing the development of pulmonary hypertension induced by MCT. The ability of SLNs to improve the sustained release property of RF-22c and to direct it to the target site may be therapeutically advantageous for PAH patients. In particular, SLN could be able to ensure high concentrations of RF22-c around damaged arteries and thus get a significant therapeutic efficacy in PAH patients with a lower frequency of administration. However, further studies will be necessary to deepen the unique properties of this new formulation, thus providing the basis for new therapeutic strategies in PAH that exploit 5-LO target and nanotherapeutic delivery system.

## Data Availability Statement

All datasets generated for this study are included in the article/supplementary material.

## Ethics Statement

The animal study was reviewed and approved by Stabulario Centralizzato d'Ateneo of the University of Campania Luigi Vanvitelli.

## Author Contributions

Conceptualization: BD’A, AL, RE. Data curation GS, AL, CP. Funding acquisition: BD’A, AM-R. Investigation: GS, AL, DS, FB, AM-R, JX. Supervision: BD’A, LB, RF, CP. Validation: GS, CP, RF. Writing—original draft: RE, AL, RF, GS, CP. Writing—review and editing: BD’A, LB, CP, RF.

## Funding

This work was supported by PRIN 2015 no. 201532AHAE_004 from the Italian Ministry of Education, University and Research (MIUR).

## Conflict of Interest

The authors declare that the research was conducted in the absence of any commercial or financial relationships that could be construed as a potential conflict of interest.

## References

[B1] Al-Husseini A. Wijesinghe D. S. Farkas L. Kraskauskas D. Drake J. I. Van-Tassel B. (2015). Increased eicosanoid levels in the sugen/chronic hypoxia model of severe pulmonary hypertension. PloS One 10, e012015. 10.1371/journal.pone.0120157 PMC4364907 25785937

[B2] Bittleman D. B. Casale T. B. (1995). 5-Hydroxyeicosatetraenoic acid (HETE)-induced neutrophil transcellular migration is dependent upon enantiomeric structure. Am. J. Respir. Cell. Mol. Biol. 12, 260–267. 10.1165/ajrcmb.12.3.7873191 7873191

[B3] Brugè F. Damiani E. Puglia C. Offerta A. Armeni T. Littarru G. P. (2013). Nanostructured lipid carriers loaded with CoQ10: effect on human dermal fibroblasts under normal and UVA-mediated oxidative conditions. Int. J. Pharm. 455, 348–356. 10.1016/j.ijpharm.2013.06.075 23850626

[B4] Bruno F. Spaziano G. Liparulo A. Roviezzo F. Nabavi S. M. Sureda A. (2018). Recent advances in the search for novel 5-lipoxygenase inhibitors for the treatment of asthma. Eur. J. Med. Chem. 153, 65–72. 10.1016/j.ejmech.2017.10.020 29133059

[B5] Cappetta D. De Angelis A. Spaziano G. Tartaglione G. Piegari E. Esposito G. (2018). Lung mesenchymal stem cells ameliorate elastase-induced damage in an animal model of emphysema. Stem Cells Int. 14, 9492038. 10.1155/2018/9492038 PMC5872595 29731780

[B6] Corris P. Degano B. (2014). Severe pulmonary arterial hypertension: treatment options and the bridge to transplantation. Eur. Respir. Rev. 23, 488–497. 10.1183/09059180.00007214 25445947 PMC9487408

[B7] Cui B. Cheng Y. S. Dai D. Z. Li N. Zhang T. T. Dai Y. (2009). CPU0213, a non-selective ETA/ETB receptor antagonist, improves pulmonary arteriolar remodeling of monocrotaline-induced pulmonary hypertension in rats. Clin. Exp. Pharmacol. Physiol. 36 (2), 169–175. 10.1111/j.1440-1681.2008.05044 18986320

[B8] Dorfm̈ller P. Perros F. Balabanian K. Humbert M. (2003). Inflammation in pulmonary arterial hypertension. Eur. Respir. J. 22, 358–363. 10.1183/09031936.03.00038903 12952274

[B9] Du Y. Plante E. Janicki J. S. Brower G. L. (2010). Temporal evaluation of cardiac myocyte hypertrophy and hyperplasia in male rats secondary to chronic volume overload. Am. J. Pathol. 177 (3), 1155–1163. 10.2353/ajpath.2010.090587 20651227 PMC2928950

[B10] Fallah F. (2015). Recent strategies in treatment of pulmonary arterial hypertension, a review. Glob. J. Health Sci. 7, 307–322. 10.5539/gjhs.v7n4p307 25946920 PMC4802183

[B11] Filosa R. Peduto A. Aparoy P. Schaible A. M. Luderer S. Krauth V. (2013). Discovery and biological evaluation of novel 1,4-benzoquinone and related resorcinol derivatives that inhibit 5-lipoxygenase. Eur. J. Med. Chem. 67, 269–279. 10.1016/j.ejmech.2013.06.039 23871907

[B12] Filosa R. Peduto A. Schaible A. M. Krauth V. Weinigel C. Barz D. (2015). Novel series of benzoquinones with high potency against 5-lipoxygenase in human polymorphonuclear leukocytes. Eur. J. Med. Chem. 94, 132–139. 10.1016/j.ejmech.2015.02.042 25765759

[B13] Geszke-Moritz M. Moritz M. (2016). Solid lipid nanoparticles as attractive drug vehicles: Composition, properties and therapeutic strategies. Mater Sci. Eng. C. Mater Biol. Appl. 68, 982–994. 10.1016/j.msec.2016.05.119 27524099

[B14] Goethals E. C. Elbaz A. Lopata A. L. Bhargava S. K. Bansal V. (2013). Decoupling the effects of the size, wall thickness, and porosity of curcumin-loaded chitosan nanocapsules on their anticancer efficacy: size is the winner. Langmuir 29, 658–666. 10.1021/la3033836 23078204

[B15] Hakim T. S. Sugimori K. Ferrario L. (1996). Analysis of the double occlusion which provides four pressure gradients. Eur. Respir. J. 9, 2578–2583. 10.1183/09031936.96.09122578 8980972

[B16] Hassoun P. M. Mouthon L. Barberà J. A. Eddahibi S. Flores S. C. Grimminger F. (2009). Inflammation, growth factors, and pulmonary vascular remodeling. J. Am. Coll. Cardiol. 54, S10–S19. 10.1016/j.jacc.2009.04.006 19555853

[B17] Ishihara T. Hayashi E. Yamamoto S. Kobayashi C. Tamura Y. Sawazaki R. (2015). Encapsulation of beraprost sodium in nanoparticles: analysis of sustained release properties, targeting abilities and pharmacological activities in animal models of pulmonary arterial hypertension. J. Control Release. 197, 97–104. 10.1016/j.jconrel.2014.10.029 25449809

[B18] Itoh T. Nagaya N. Ishibashi-Ueda H. Kyotani S. Oya H. Sakamaki F. (2006). Increased plasma monocyte chemoattractant protein-1 level in idiopathic pulmonary arterial hypertension. Respirology 11, 158–163. 10.1111/j.1440-1843.2006.00821.x 16548900

[B19] Jones J. E. Walker J. L. Song Y. Weiss N. Cardoso W. V. Tuder R. M. (2004). Effect of 5-lipoxygenase on the development of pulmonary hypertension in rats. Am. J. Physiol. Heart Circ. Physiol. 286, 1775–1784. 10.1152/ajpheart.00281.2003 14726295

[B20] Kimura H. Okada O. Tanabe N. Tanaka Y. Terai M. Takiguchi Y. (2001). Plasma monocyte chemoattractant protein-1 and pulmonary vascular resistance in chronic thromboembolic pulmonary hypertension. Am. J. Respir. Crit. Care Med. 164, 319–324. 10.1164/ajrccm.164.2.2006154 11463608

[B21] Marsh L. M. Jandl K. Grünig G. Foris V. Bashir M. Ghanim B. (2018). The inflammatory cell landscape in the lungs of patients with idiopathic pulmonary arterial hypertension. Eur. Respir. J. 25, 51. 10.1183/13993003.01214-2017 PMC6383570 29371380

[B22] Meyrick B. Gamble W. Reid L. (1980). Development of Crotalaria pulmonary hypertension: hemodynamic and structural study. Am. J. Physiol. 239, 692–702. 10.1152/ajpheart.1980.239.5.H692 6449154

[B23] Montani D. Günther S. Dorfmüller P. Perros F. Girerd B. Garcia G. (2013). Pulmonary arterial hypertension. Orphanet J. Rare Dis. 8, 97. 10.1186/1750-1172-8-97 23829793 PMC3750932

[B24] Mori T. A. Beilin L. J. (2004). Omega-3 fatty acids and inflammation. Curr. Atheroscler Rep. 6, 461–467. 10.1007/s11883-004-0087-5 15485592

[B25] Nicolls M. R. Taraseviciene-Stewart L. Rai P. R. Badesch D. B. Voelkel N. F. (2005). Autoimmunity and pulmonary hypertension: a perspective. Eur. Respir. J. 26, 1110–1118. 10.1183/09031936.05.00045705 16319344

[B26] Nogueira-Ferreira R. Vitorino R. Ferreira R. Henriques-Coelho T. (2015). Exploring the monocrotaline animal model for the study of pulmonary arterial hypertension: a network approach. Pulm. Pharmacol. Ther. 35, 8–16. 10.1016/j.pupt.2015.09.007 26403584

[B27] Peduto A. Scuotto M. Krauth V. Roviezzo F. Rossi A. Temml V. (2017). Optimization of benzoquinone and hydroquinone derivatives as potent inhibitors of human 5-lipoxygenase. Eur. J. Med. Chem. 127, 715–726. 10.1016/j.ejmech.2016.10.046 27836196

[B28] Petronzi C. Filosa R. Peduto A. Monti M. C. Margarucci L. Massa A. (2011). Structure-based design, synthesis and preliminary anti-inflammatory activity of bolinaquinone analogues. Eur. J. Med. Chem. 46, 488–496. 10.1016/j.ejmech.2010.11.028 21163556

[B29] Petronzi C. Festa M. Peduto A. Castellano M. Marinello J. Massa A. (2013). Cyclohexa-2,5-diene-1,4-dione-based antiproliferative agents: design, synthesis, and cytotoxic evaluation. J. Exp. Clin. Cancer Res. 32, 24. 10.1186/1756-9966-32-24 23631805 PMC3666920

[B30] Price L. C. Wort S. J. Perros F. Dorfmuller P. Huerta A. Montani D. (2012). Inflammation in pulmonary arterial hypertension. Chest 141, 210–221. 10.1378/chest.11-0793 22215829

[B31] Puglia C. Rizza L. Drechsler M. Bonina F. (2010). Nanoemulsions as vehicles for topical administration of glycyrrhetic acid: characterization and *in vitro* and *in vivo* evaluation. Drug Deliv. 17, 123–129. 10.3109/10717540903581679 20136625

[B32] Puglia C. Frasca G. Musumeci T. Rizza L. Puglisi G. Bonina F. (2012). Curcumin loaded NLC induces histone hypoacetylation in the CNS after intraperitoneal administration in mice. Eur. J. Pharm. Biopharm. 81, 288–293. 10.1016/j.ejpb.2012.03.015 22504443

[B33] Puglia C. Offerta A. Tirendi G. G. Tarico M. S. Curreri S. Bonina F. (2016). Design of solid lipid nanoparticles for caffeine topical administration. Drug Deliv. 23 (1), 36–40. 10.3109/10717544.2014.903011 24735249

[B34] Puglia C. Blasi P. Ostacolo C. Sommella E. Bucolo C. Platania C. M. (2018). Innovative nanoparticles enhance N-Palmitoylethanolamide intraocular delivery. Front. Pharmacol. 9, 285. 10.3389/fphar.2018.00285 29643808 PMC5882782

[B35] Qian J. Tian W. Jiang X. Tamosiuniene R. Sung Y. K. Shuffle E. M. (2015). Leukotriene B4 activates pulmonary artery adventitial fibroblasts in pulmonary hypertension. Hypertension 66, 1227–1239. 10.1161/HYPERTENSIONAHA.115.06370 26558820 PMC4646718

[B36] Rabinovitch M. (2012). Molecular pathogenesis of pulmonary arterial hypertension. J. Clin. Invest. 122, 4306–4313. 10.1172/JCI60658 23202738 PMC3533531

[B37] Sanchez O. Marcos E. Perros F. Fadel E. Tu L. Humbert M. (2007). Role of endothelium-derived CC chemokine ligand 2 in idiopathic pulmonary arterial hypertension. Am. J. Respir. Crit. Care Med. 176, 1041–1047. 10.1164/rccm.200610-1559OC 17823354

[B38] Schaible A. M. Trabe H. Temml V. Noha S. M. Filosa R. Peduto A. (2013). Potent inhibition of human 5-lipoxygenase and microsomal prostaglandin E2 synthase-1 by the anti-carcinogenic and anti-inflammatory agent embelin. Biochem. Pharmacol. 86, 476–486. 10.1016/j.bcp.2013.04.015 23623753

[B39] Schaible A. M. Filosa R. Temml V. Krauth V. Matteis M. Peduto A. (2014). Elucidation of the molecular mechanism and the efficacy *in vivo* of a novel 1,4-benzoquinone that inhibits 5-lipoxygenase. Br. J. Pharmacol. 171, 2399–2412. 10.1111/bph.12592 24467325 PMC3997279

[B40] Schaible A. M. Filosa R. Krauth V. Temml V. Pace S. Garscha U. (2016). The 5-lipoxygenase inhibitor RF-22c potently suppresses leukotriene biosynthesis in cellulo and blocks bronchoconstriction and inflammation *in vivo* . Biochem. Pharmacol. 112, 60–71. 10.1016/j.bcp.2016.04.019 27157409

[B41] Sharma S. Ruffenach G. Umar S. Motayagheni N. Reddy S. T. Eghbali M. (2016). Role of oxidized lipids in pulmonary arterial hypertension. Pulm. Circ. 6 (3), 261–273. 10.1086/687293 27683603 PMC5019079

[B42] Singh S. R. Sullo N. Matteis M. Spaziano G. McDonald J. Saunders R. (2016). Nociceptin/orphanin FQ (N/OFQ) modulates immunopathology and airway hyperresponsiveness representing a novel target for the treatment of asthma. Br. J. Pharm. 173, 1286–1301. 10.1111/bph.13416 PMC4940820 26756419

[B43] Song Y. Jones J. E. Beppu H. Keaney J. F. Jr. Loscalzo J. Zhang Y. Y. (2005). Increased susceptibility to pulmonary hypertension in heterozygous BMPR2-mutant mice. Circulation 112, 553–562. 10.1161/CIRCULATIONAHA.104.492488 16027259 PMC1472405

[B44] Stenmark K. R. Morganroth M. L. Remigio L. K. Voelkel N. F. Murphy R. C. Henson P. M. (1985). Alveolar inflammation and arachidonate metabolism in monocrotaline-induced pulmonary hypertension. Am. J. Physiol. 248, 859–866. 10.1152/ajpheart.1985.248.6.H859 3923843

[B45] Tabata T. Ono S. Song C. Noda M. Suzuki S. Tanita T. (1997). Role of leukotriene B4 in monocrotaline-induced pulmonary hypertension. Nihon Kyobu Shikkan Gakkai Zasshi 35 (2), 160–166. 9103852

[B46] Tamosiuniene R. Tian W. Dhillon G. Wang L. Sung Y. K. Gera L. (2011). Regulatory T cells limit vascular endothelial injury and prevent pulmonary hypertension. Circ. Res. 109, 867–879. 10.1161/CIRCRESAHA.110.236927 21868697 PMC3204361

[B47] Taraseviciene-Stewart L. Nicolls M. R. Kraskauskas D. Scerbavicius R. Burns N. Cool C. (2007). Absence of T cells confers increased pulmonary arterial hypertension and vascular remodeling. Am. J. Respir. Crit. Care Med. 175, 1280–1289. 10.1164/rccm.200608-1189OC 17413127 PMC2176089

[B48] Tian W. Jiang X. Tamosiuniene R. Sung Y. K. Qian J. Dhillon G. (2013). Blocking macrophage leukotriene b4 prevents endothelial injury and reverses pulmonary hypertension. Sci. Transl. Med. 5 (200), 200ra117. 10.1126/scitranslmed.3006674 PMC4016764 23986401

[B49] Tong Y. Jiao Q. Liu Y. Lv J. Wang R. Zhu L. (2018). Maprotiline prevents monocrotaline-induced pulmonary arterial hypertension in rats. Front. Pharmacol. 9:1032. 10.3389/fphar.2018.01032 30298002 PMC6160570

[B50] Tuder R. M. Voelkel N. F. (1998). Pulmonary hypertension and inflammation. J. Lab. Clin. Med. 132, 16–24. 10.1016/S0022-2143(98)90020-8 9665367

[B51] Uhlig S. Wollin L. (1994). An improved setup for the isolated perfused rat lung. J. Pharmacol. Toxicol. Methods 31, 85–94. 10.1016/1056-8719(94)90047-7 8032099

[B52] Uhlig S. Brasch F. Wollin L. Fehrenbach H. Richter J. Wendel A. (1995). Functional and fine structural changes in isolated rat lungs challenged with endotoxin ex vivo and in vitro. Am. J. Pathol. 146, 1235–1247. 7747816 PMC1869288

[B53] Voelkel N. F. Tuder R. M. Wade K. Hoper M. Lepley R. A. Goulet J. L. (1996). Inhibition of 5-lipoxygenase-activating protein (FLAP) reduces pulmonary vascular reactivity and pulmonary hypertension in hypoxic rats. J. Clin. Invest. 97, 2491–2498. 10.1172/JCI118696 8647941 PMC507334

[B54] Wright L. Tuder R. M. Wang J. Cool C. D. Lepley R. A. Voelkel N. F. (1998). 5-Lipoxygenase and 5-lipoxygenase activating protein (FLAP) immunoreactivity in lungs from patients with primary pulmonary hypertension. Am. J. Respir. Crit. Care Med. 157, 219–229. 10.1164/ajrccm.157.1.9704003 9445303

[B55] Zabini D. Heinemann A. Foris V. Nagaraj C. Nierlich P. Balint Z. (2014). Comprehensive analysis of inflammatory markers in chronic thromboembolic pulmonary hypertension patients. Eur. Respir. J. 44, 951–962. 10.1183/09031936.00145013 25034560

[B56] Zappavigna S. Scuotto M. Cossu A. M. Ingrosso D. De Rosa M. Schiraldi C. (2016). The 1,4 benzoquinone-featured 5-lipoxygenase inhibitor RF-Id induces apoptotic death through downregulation of IAPs in human glioblastoma cells. J. Exp. Clin. Cancer Res. 35, 167. 10.1186/s13046-016-0440-x 27770821 PMC5075202

